# DNA extraction approaches substantially influence the assessment of the human breast milk microbiome

**DOI:** 10.1038/s41598-019-55568-y

**Published:** 2020-01-10

**Authors:** Chloe A. Douglas, Kerry L. Ivey, Lito E. Papanicolas, Karen P. Best, Beverly S. Muhlhausler, Geraint B. Rogers

**Affiliations:** 1 South Australian Health and Medical Research Institute, Healthy Mothers, Babies and Children Theme, Women’s and Children’s Hospital, King William Road, Adelaide, South Australia Australia; 2 South Australian Health and Medical Research Institute, Microbiome & Host Health Programme, Adelaide, South Australia Australia; 3 Faculty of Health Science, Discipline of Medicine – Paediatrics and Reproductive Health, The University of Adelaide, Adelaide, South Australia Australia; 4 College of Medicine and Public Health, Flinders University, Adelaide, South Australia Australia; 5 Nutrition & Health Program, Health and Biosecurity, CSIRO, Adelaide, South Australia Australia; 6 Department of Food and Wine Science, School of Agriculture, Food and Wine, The University of Adelaide, Adelaide, South Australia Australia; 7 Harvard T.H. Chan School of Public Health, Department of Nutrition, Boston, Massachusetts USA

**Keywords:** Microbiome, Bioinformatics, RNA sequencing, Bacteria

## Abstract

In addition to providing nutritional and bioactive factors necessary for infant development, human breast milk contains bacteria that contribute to the establishment of commensal microbiota in the infant. However, the composition of this bacterial community differs considerably between studies. We hypothesised that bacterial DNA extraction methodology from breast milk samples are a substantial contributor to these inter-study differences. We tested this hypothesis by applying five widely employed methodologies to a mock breast milk sample and four individual human breast milk samples. Significant differences in DNA yield and purity were observed between methods (P < 0.05). Microbiota composition, assessed by 16S rRNA gene amplicon sequencing, also differed significantly with extraction methodology (P < 0.05), including in the contribution of contaminant signal. Concerningly, many of the bacterial taxa identified here as contaminants have been reported as components of the breast milk microbiome in other studies. These findings highlight the importance of using stringent, well-validated, DNA extraction methodologies for analysis of the breast milk microbiome, and exercising caution interpreting microbiota data from low-biomass contexts.

## Introduction

In addition to nutrients and bioactive components, human breast milk contains bacteria^1^, commonly referred to as the breast milk microbiome. These bacterial populations are thought to contribute to the establishment of commensal bacterial communities in the infant, as well as supporting the maturation of the gut, and development of the immune system^1,2^. The potential importance of microbes introduced with breast milk on early-life development has led to increasing efforts to accurately define them. Early culture- and PCR-based methods, predominantly reported the presence of members of the *Staphylococcus*, *Streptococcus*, *Lactobacillus* and *Bifidobacterium* genera^3–12^. More recently, 16S rRNA gene amplicon sequencing has become an increasingly popular approach to characterising the bacterial content of breast milk samples^13–33^.

By enabling the detection of species that are refractory to standard culture methodologies, as well as providing insight into the relative abundance of constitutive bacteria, 16S rRNA gene sequencing offers substantial analytical advantages. However, the composition of the breast milk microbiome reported in sequencing-based studies varies substantially. For example, while two studies have described a “core” human breast milk microbiome (bacteria that are present in all samples analysed) from healthy mothers, consisting of 7 and 9 taxa respectively, only three of these taxa were present in both (*Propionibacterium, Staphylococcus*, and *Streptococcus)*^27,34^. Notably, many of the other bacterial taxa often reported as part of the breast milk microbiome, including *Delftia*^15,16,22,23,32^, *Flavobacterium*^18,24,35^, *Pseudomonas*^13–19,21–23,27,34^, *Burkholderia*^19,21,25,32^, *Sphingomonas*^15,16,20,22,24,27,31,34^, *Corynebacterium*^13–15,17,23,24,27,28,34,35^ and *Propionibacterium*^14,16,17,22,26,31^, are commonly identified as reagent contaminants in low biomass samples^36–39^. Given that the biomass of breast milk is typically low^12^, it is logical to question whether these bacteria are truly part of the breast milk microbiome, or represent reagent contamination.

One of the main differences in the analytical protocols used in published breast milk microbiome studies is the methodology used for bacterial DNA extraction. To extract DNA, bacterial cells within the sample must be lysed effectively (achieved variously through thermal, chemical, enzymatic, or mechanical cell disruption), the DNA released and separated from cell debris and PCR inhibitors. The methodology must be sufficiently stringent to efficiently lyse both Gram negative and Gram positive cells^40,41^, to prevent distortion of the relative abundance of the species present and to provide sufficient DNA yield to limit the contribution of signal from reagent contaminants^42^. Differences in extraction methodologies have been shown previously to result in significant differences in sequencing data^43,44^, including in the analysis of stool, saliva, and vaginal samples^45–48^. However, no assessment of microbiota variation arising as a result of differences in DNA extraction methodologies has been conducted in relation to breast milk.

The aim of our study was to determine how five commonly employed approaches to bacterial DNA extraction influence the observed microbiota composition in breast milk samples, as assessed by 16S rRNA gene sequencing. Four methods based on commercial DNA extraction kits (DNeasy PowerLyzer PowerSoil DNA Isolation, Sigma-Aldrich GenElute Bacterial Genomic DNA Kit, QIAamp DNA Stool Mini Kit, and QIAamp DNA Stool Mini Kit with bead beating) and a manual phenol-chloroform-based method. These extraction methods were chosen on the basis of previous use in breast milk extraction studies, employing a range of different lysis methods, and previous use in low biomass sample. Each method was applied to a mock breast milk sample (a defined bacterial community added to filter-sterilised breast milk) and to aliquots of human breast milk samples. Relative performance and reproducibility was assessed based on DNA yield and purity, and bacterial community composition.

## Results

### DNA yield and purity

**Figure 1:**
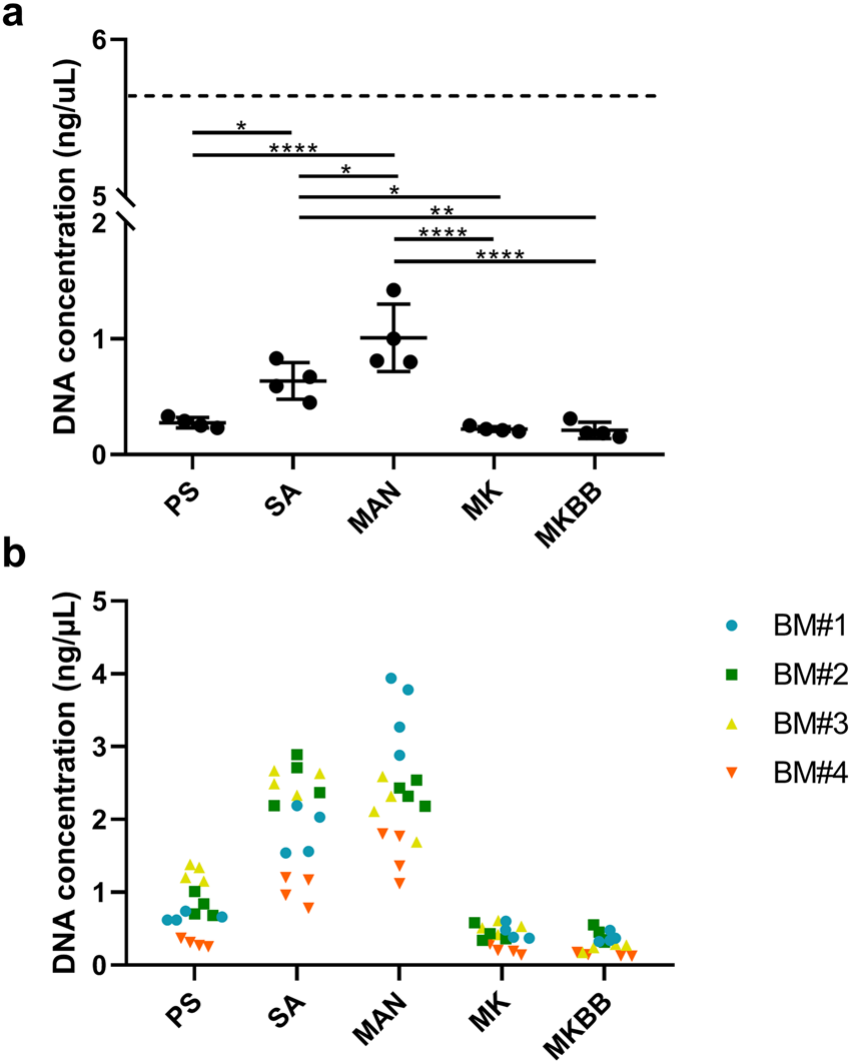
DNA concentration (ng/µL) of mock breast milk (**a**) and human breast milk samples (**b**) as determined by Qubit dsDNA HS Assay kit. The dashed line represents the expected DNA concentration of 5.49 ng/uL, based on the bacteria added to the mock breast milk. Expected concentration was calculated based on the size of each bacterial genome, the weight of a base pair, and the CFU of each bacteria. *P < 0.05, **P < 0.01, ***P < 0.001, ****P < 0.0001. PS = DNeasy PowerLyzer PowerSoil DNA Isolation, SA = Sigma-Aldrich GenElute Bacterial Genomic DNA Kit, MAN = manual phenol-chloroform extraction method, MK = QIAamp DNA Stool Mini Kit, MKBB = QIAamp DNA Stool Mini Kit with bead beating.

Comparison of DNA yield and purity was based on extractions from mock breast milk samples. Yield differed significantly between methods (ANOVA, P < 0.001, Fig. 1a) and was significantly higher for Sigma-Aldrich GenElute (Mean ± SD, 0.635 ± 1.6 ng/µL) and manual phenol-chloroform (1.01 ± 0.29 ng/µL), compared to the other methodologies (Tukey’s HSD, P < 0.05). Extract purity, as assessed by 260 nm/280 nm absorbance ratios, also differed significantly between methods (ANOVA, P = 0.005), with mean ratios achieved with QIAamp DNA stool Mini Kit plus bead beating significantly higher than those achieved using other methods (Tukey’s HSD, P < 0.05).

### Bacterial DNA amplification and sequencing depth

Assessment of DNA amplification and sequencing depth were also performed on extractions from mock breast milk samples. 16S rRNA gene qPCR yield differed significantly between extraction methods (ANOVA, P < 0.001), with those obtained using manual phenol-chloroform extractions significantly higher than achieved from all other methods (Tukey’s HSD, P < 0.05). 16S rRNA gene sequencing depth also differed significantly between extraction methods (ANOVA, P = 0.008), with significantly more reads achieved for extracts obtained using DNeasy PowerLyzer PowerSoil DNA Isolation (Mean reads = 9368.75, SD = 3000.1) compared to QIAamp DNA stool Mini Kit and QIAamp DNA stool Mini Kit with bead beating (P < 0.05) (Fig. S1A).

### Microbiota composition

**Figure 2:**
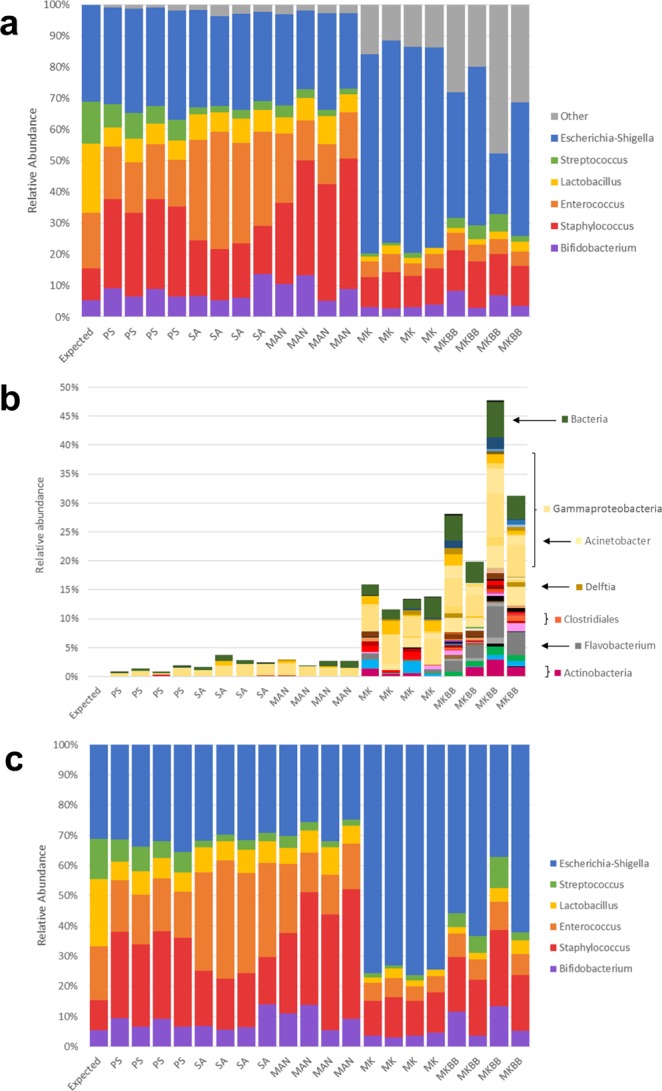
Mock breast milk relative abundance output across methods and repeats compared to expected. Mock breast milk relative abundance (**a**), spurious bacteria not added into mock (**b**), adjusted abundance with spurious bacteria removed (**c**). PS = DNeasy PowerLyzer PowerSoil DNA Isolation, SA = Sigma-Aldrich GenElute Bacterial Genomic DNA Kit, MAN = manual phenol-chloroform extraction method, MK = QIAamp DNA Stool Mini Kit, MKBB = QIAamp DNA Stool Mini Kit with bead beating.

Because the mock breast milk sample contained a defined ratio of known species, it was possible to predict the contribution of each taxon to 16S rRNA gene sequencing profiles based on their relative abundance and rRNA operon number. When 16S rRNA gene sequencing profiles were compared to predicted composition, relative abundances of *Bifidobacterium, Staphylococcus*, *Enterococcus*, *Lactobacillus* and *Escherichia-Shigella* did not differ significantly between any of the kits when compared to the expected relative abundances after correction for multiple comparison (Fig. 2a). *Streptococcus*, however, was significantly underrepresented by QIAamp DNA Stool Mini Kit (Dunn’s Test, P < 0.05).

**Figure 3:**
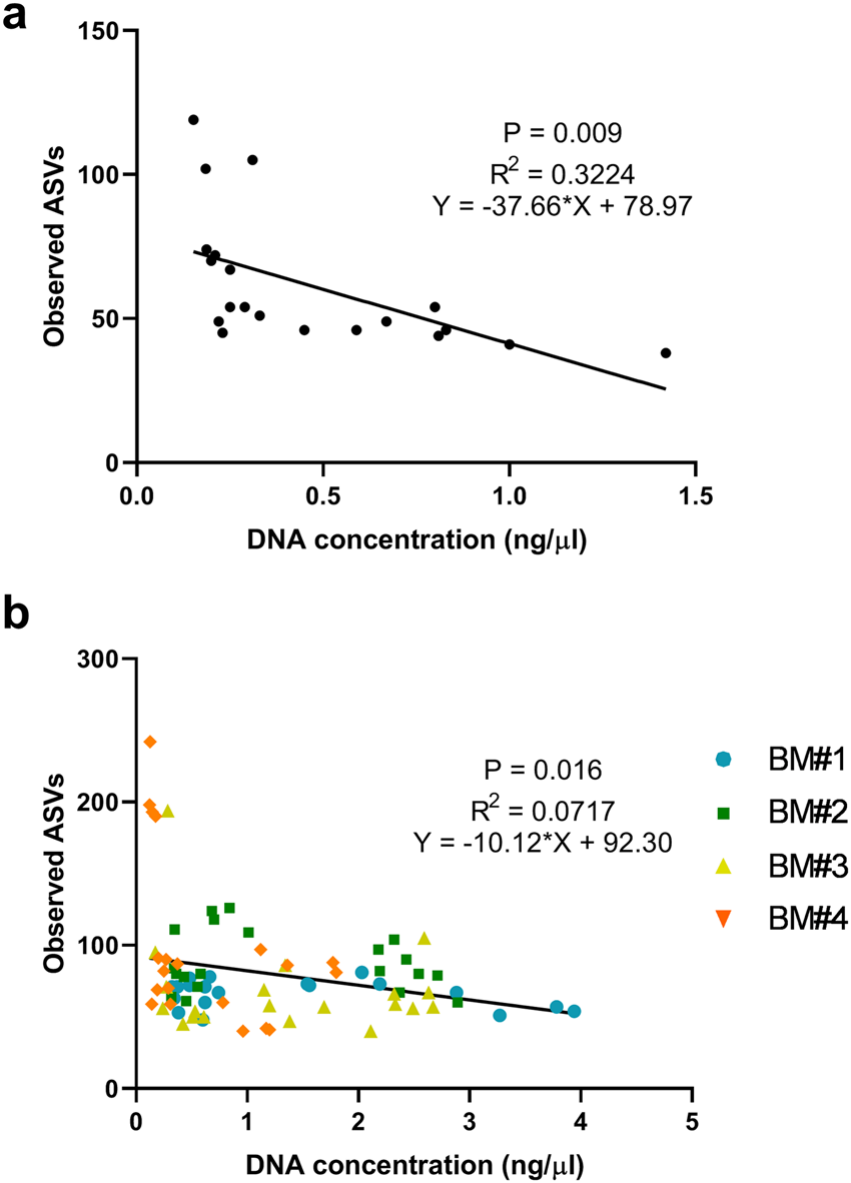
Linear regression analysis of DNA concentrations (ng/µL) and observed ASVs in mock breast milk samples (**a**) and human breast milk samples (**b**).

All extraction methodologies also resulted in the detection of bacterial taxa that were not present in the mock breast milk sample (Fig. 2b). Two methods resulted in a substantial portion of sequence reads representing contaminant taxa, with spurious ASVs accounting for up to 47.7% of taxon relative abundance when extraction was performed using the QIAamp DNA Stool Mini Kit with bead beating (mean of 31.7% across the 4 repeats) (Fig. 2b). The QIAamp DNA Stool Mini Kit also returned a high number of spurious ASVs accounting for up to 15.8% of taxon relative abundance (mean of 13.6% across the 4 repeats). The three remaining methods (DNeasy PowerLyzer PowerSoil DNA Isolation, Sigma-Aldrich GenElute Bacterial Genomic DNA Kit and manual phenol-chloroform) returned a spurious signal accounting for <5% relative abundance. Detection of a spurious signal and DNA concentration showed a significant inverse relationship (P = 0.009) (Fig. 3a).

A total of 121 ASVs, representing ten different phyla, were detected, despite not being present in the mock breast milk sample (Fig. S2). Of these, fifteen belonged to Gammaproteobacteria, accounting for a 13.8% of the total relative abundance in mock breast milk sample repeats extracted with QIAamp DNA Stool Mini Kit with bead beating. Within this phylum, *Delftia* and *Acinetobacter* were detected at high relative abundance and present in all mock breast milk sample repeats from this method, averaging 2.7% and 2.3% relative abundance respectively. Twenty-nine spurious Bacteroidetes ASVs accounted for 5.9% of the relative abundance in the mock breast milk sample extracted with QIAamp DNA Stool Mini Kit with bead beating, with one bacteria, *Flavobacterium*, present in all repeats and accounting for an average of 3.3% relative abundance.

The removal of spurious reads and re-scaling of sequence data did not remove intergroup differences in microbiota composition (Fig. 2c). In particular, an over-representation of *Escherichia-Shigella* and an under-representation of taxa other than *Staphylococcus* remained evident for QIAamp DNA Stool Mini Kit and QIAamp DNA Stool Mini Kit with bead beating methods.

### Analysis of human breast milk samples

Assessments based on mock breast milk samples indicated that extraction methodology significantly influenced the DNA yield, purity, and 16S rRNA gene sequencing output, with suboptimal extraction resulting in distorted microbial composition and a high levels of spurious signal. Whether comparable effects could be identified when these methodologies were applied to aliquots of real breast milk was then investigated.

When the five DNA extraction methodologies were applied to aliquots of four human breast milk samples, the trends observed for the mock sample were replicated. Again, significant differences were observed in DNA yield between methods (Kruskal-Wallis ANOVA, P < 0.001, Fig. 1b), with post-hoc analysis revealing significantly higher yields from the phenol-chloroform based method compared to QIAamp DNA stool Mini Kit and bead beating in three samples (BM1, BM3 and BM4, Dunn’s Test, P < 0.01). In the case of two of the samples, significant differences in DNA extract purity were also observed (BM1 and BM4, ANOVA, P < 0.001), with QIAamp DNA stool Mini Kit plus bead beating producing significantly higher purity that that achieved using other methods (Tukey’s HSD, P < 0.05). Sequencing depth also differed significantly with extraction method in two of the human breast milk samples (BM3 and BM4, Kruskal-Wallis ANOVA with Dunn’s Test, P < 0.05) (Fig. S1B). Alpha-diversity measures differed significantly between kits in some of the breast milk samples but the same trends were not observed across all samples (Fig. S3).

**Figure 4:**
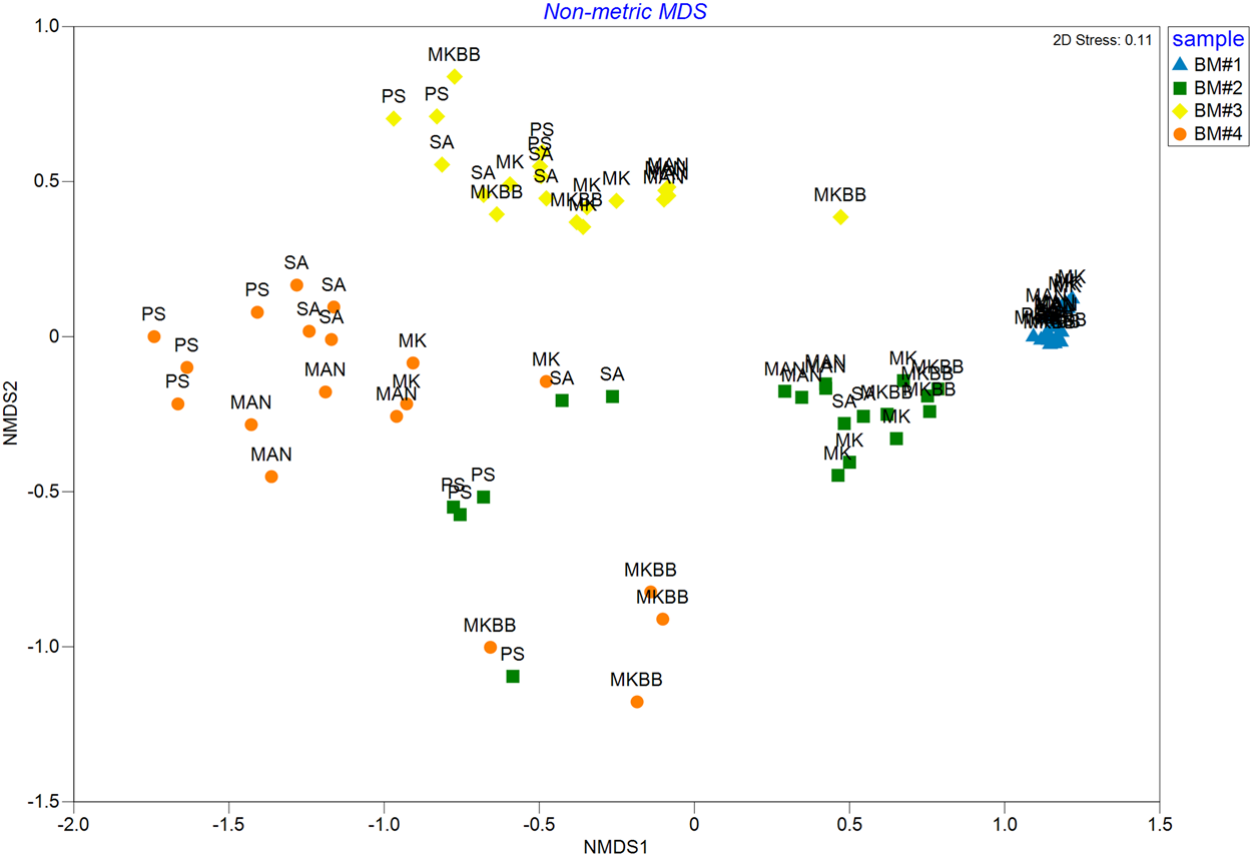
nMDS plot of Weighted UniFrac distance matrix for all human breast milk samples and repeats across methods. PERMANOVA revealed significant differences between samples (Pseudo-F = 109.47, P = 0.0001) and between methods (Pseudo-F = 15.162, P = 0.0001). Samples are indicated by colour and method by abbreviation. PS = DNeasy PowerLyzer PowerSoil DNA Isolation, SA = Sigma-Aldrich GenElute Bacterial Genomic DNA Kit, MAN = manual phenol-chloroform extraction method, MK = QIAamp DNA Stool Mini Kit, MKBB = QIAamp DNA Stool Mini Kit with bead beating.

Human breast milk samples were largely dominated by *Staphylococcus* and/or *Streptococcus* and/or *Pseudomonas*. However, significant differences in breast milk microbiota composition were observed (Fig. 4). Variation in microbiota dispersion were associated with both sample donor and DNA extraction methodology (P(perm) = 0.0001). Based on Pseudo-F scores, inter-sample differences (Pseudo-F = 109.47) were a greater contributor to variation in composition than extraction method (Pseudo-F = 15.162). Regardless of the methodology used, microbiota profiles derived from repeat extractions of individual samples did not differ significantly (P = 0.755).

Bacterial taxa identified as contaminants in the mock breast milk sample, including members of the *Pseudomonas*, *Delftia* and *Flavobacterium* genera, were also detected in the human breast milk. *Pseudomonas* relative abundance varied substantially between human breast milk samples, dominating BM1 but scarcely present in BM2, BM3 and BM4. *Delftia* and *Flavobacterium* accounted for up to 9% and 6% of the relative abundance respectively and were present in all breast milk samples but were not detected across all methods and repeats.

### Relationship between extraction methodology and artefactual bacterial detection

In addition to the potential to distort the taxon relative abundance due to differential lysis, suboptimal DNA extraction also appears to be a substantial contributor to the risk of artefactual bacterial detection, most likely because of its impact on DNA concentration. This is particularly problematic in the analysis of breast milk samples, where there is overlap between the species that have been isolated from breast milk by culture, and those that commonly appear as sequencing artefacts (for example, *Streptococcus*). However, it is possible to assess the likelihood that a given ASV is a contaminant based on the relationship between their relative abundance and DNA concentration (contaminants become relatively more prevalent where there is reduced bacterial template for PCR amplification). Therefore, a further analysis was performed to assess the relationship between *Pseudomonas*, the most prevalent potential contaminant taxon, and DNA concentration.

Indeed, a significant inverse relationship was identified between ASV richness and DNA concentration in the mock breast milk sample (Fig. S4A). Taxa detected, but not included, in the mock breast milk sample, also showed this relationship (Fig. S4B–D). In contrast, the relative abundance of genuine constituents, such as *Streptococcus* and *Staphylococcus*, showed a positive or null correlation with DNA concentration (Fig. S4E,F).

When the distribution of these same taxa was examined in microbiota profiles generated from human breast milk samples, similar patterns were observed (Fig. S5). A significant inverse relationship was identified between ASV richness and DNA concentration (Fig. 3b). In particular, relative abundance of *Delftia* and *Flavobacterium*, which were identified as spurious taxa in the mock breast milk sample, demonstrated the same inverse relationship with DNA concentration (Fig. S5A,B). In contrast, *Streptococcus* and *Staphylococcus*, which is commonly isolated from human breast milk samples by culture, showed the same positive or null correlation with DNA concentration as observed in the mock sample (Fig. S5D,E). *Pseudomonas* relative abundance did not, however, behave the same in the human breast milk samples as in the mock breast milk (Fig. S5C). The relationship between relative abundance and DNA concentration was demonstrated a positive or null correlation with DNA concentration, whereas with the mock breast milk it demonstrated an inverse relationship. This suggests that while the spurious detection of *Pseudomonas* can occur at low DNA concentrations, its presence in human breast milk may also be genuine.

## Discussion

The potential for the bacterial content of breast milk to contribute to the establishment of the early life commensal microbiota, and by extension, immune and metabolic homeostasis, has been much discussed^5,13,26^. Efforts to characterise this bacterial content through 16S rRNA gene sequencing have shown little agreement between studies^2^. Suboptimal extraction of bacterial DNA (both yield and purity) has the potential to distort the relative abundance of constituent taxa, and also to influence the contribution of artefactual bacterial signal that is considered unavoidable in the analysis of low biomass samples^37,42–48^. This study was the first to assess the impact of DNA extraction methodologies on the characterisation of human breast milk microbiota.

The assessed methodologies included a common commercial kit, QIAamp DNA Stool Mini Kit (with or without the addition of bead beating)^14,17,18,21,23,24,27–29^, DNeasy PowerLyzer PowerSoil^33^, and a high stringency phenol-chloroform method^34,35,49^, which have been used in previously published breast milk microbiome analyses. We are not aware of any published studies in which Sigma-Aldrich Gen-Elute has been used to analyse the breast milk microbiota, however, it has been used in the assessment of other low biomass samples^50^. The approaches differed particularly in regards to the mode of bacterial lysis. As predicted, the application of these methods resulted in significant differences in DNA yield and purity, 16S rRNA gene sequence read depth, and microbiota profile. The use of a mock breast milk sample, composed of defined bacterial community in filter-sterilised human breast milk, was important in allowing comparison of expected and observed metrics, and an ability to definitively identify contaminant signal. Applications of the same protocols to real breast milk samples then allowed the implications of methodological differences for breast milk microbiota characterisation to be assessed.

DNA yield differed significantly with DNA extraction methods. The use of the phenol-chloroform-based method or the Sigma-Aldrich Gen-Elute kit consistently returned significantly higher DNA concentration (also reflected in qPCR-based bacterial load estimations). However, these methods also showed the greatest intra-method variability. Furthermore, the phenol-chloroform method and the Sigma-Aldrich Gen-Elute returned the lowest purity extracts, with QIAamp DNA Stool Mini Kit and/or QIAamp DNA Stool Mini Kit with bead beating consistently returning A260/280 absorbance ratios consistent with the greatest purity. Sequence read depth differed significantly with methods, although there was no consistent trend across mock breast milk and human breast milk samples. These findings are in keeping with variations associated with different extraction strategies in other clinical contexts^45–48^.

Differences in extraction methodologies have also been associated with significant variation in microbiota profiles generated from a range of human samples^43–48^. We found this to be true for both mock and human breast milk samples. All extraction assessed methodologies resulted in an under-representation of *Lactobacillus* and *Streptococcus*, a recognised phenomenon in microbiota analysis^51^. Although all methods returned a level of spurious signal, two kit-based methods (QIAamp DNA Stool Mini Kit and QIAamp DNA Stool Mini Kit with bead beating) returned a substantial number of bacterial taxa not present in the mock breast milk sample. These overwhelmingly represented species that have been identified previously as sources of common reagent contamination in 16S rRNA gene sequencing based analysis^37^. The detection of spurious bacterial signal in both the mock breast milk and human breast milk samples was associated will low DNA yield, an interaction that that is well-established^37,52,53^, and likely to be of particular importance in the context of breast milk analysis, given the bacterial loads involved^12^. However, although PowerLyzer PowerSoil had significantly lower yield compared to phenol-chloroform and Sigma-Aldrich Gen-Elute, it returned the lowest level of spurious signal. Interestingly, the DNA extraction methods that returning low spurious signal were eluted in lab-grade water rather than supplied buffers, suggesting potential kit contamination. Our findings have important implications for how the bacterial composition of breast milk is assessed and interpreted. No single DNA extraction methodology was found to be superior in all aspects. However, an understanding of the limitations of the approaches being employed, a systematic assessment of their performance, and the ability to identify a signal that is unlikely to represent genuine bacterial sample content, are all essential if investigations of the influence of the breast milk microbiome are to yield meaningful insight.

The aim of this study was not to comprehensively assess all methodologies that might be employed to extract bacterial DNA from breast milk samples. Rather, we aimed to demonstrate that selection of an extraction methodology can have a substantial influence on the data obtained, and has the potential to result in misleading data. However, the study did have a number of limitations that must be considered. Analysis was performed on a relatively small number of breast milk samples. However, the sample size was sufficient to allow significant differences between extraction methodologies to be identified. The performance of DNA extraction methods differed somewhat between breast milk samples and the future analysis of a larger sample collection might provide insight into the characteristics that contribute to this variance. We did not attempt to definitively characterise the microbial content of breast milk samples. Arguably, this cannot be achieved using sequencing-based methods alone, but would require the additional use of culture and microscopy-based approaches to validate sequence data. In addition, we did not attempt to identify the source of spurious bacterial signal. Signal from a range of common environmental and human-associated taxa in 16S rRNA gene sequencing data from low-biomass samples is a common phenomenon that has been described widely^36–39,42^, and whether it can be completely excluded is a matter of ongoing debate. Given that the processes downstream of DNA extraction, including the sequencing reagents and methodologies, were identical, we are confident that any differences relate to the DNA extracts themselves.

In conclusion, our study highlights the critical importance of understanding the influence of methodological considerations on microbiome analysis in the context of human breast milk. It confirms the potential for widely-reported bacterial constituents of breast milk to be artefacts common to 16S rRNA gene sequencing based analysis of low biomass samples, while also supporting the genuine contribution of members of the *Staphylococcus* and/or *Streptococcus* genera to these samples.

## Methods

### Study cohort and sample collection

This study was undertaken with approval from the Southern Adelaide Clinical Human Research Ethics Committee (ORF#301.16). Breast milk samples were collected at 6 weeks post-partum from participants in a randomised controlled trial of omega-3 supplementation to breastfeeding mothers of full-term infants born vaginally (The Tummy Trial, Australian New Zealand Clinical Trials Registry, ANZCTR#12616001447448). All women provided informed consent and methods were performed in accordance with the relevant guidelines and regulations. Women were instructed to clean their breast prior to milk collection with soap and water. Before a feed, and after discarding the first few drops, women hand expressed a breast milk sample into a sterile 70 ml sample collection container. Aliquots of breast milk (1.5 mL) used in the current study were collected between May 2017 and January 2018 and were stored at −80 °C prior to analysis.

### Mock breast milk community

A pooled 6 week breast milk sample from participants in The Tummy Trial was centrifuged at 13,000 *g*, 4 °C, for 20 min (Heraeus Fresco 21, ThermoFisher Scientific, Massachusetts, USA), the fat layer removed, and the supernatant passed through a MILLEX-HP PES 0.45 µm EXPRESS filtering unit (Millipore, Cork, Ireland). Strains of bacteria commonly reported in breast milk microbiota (*Lactobacillus paracasei*, *Bifidobacterium bifidum*, *Streptococcus salivarius*, *Staphylococcus epidermidis*, *Enterococcus faecalis*, *Escherichia coli*) were cultured from clinical isolates and ATCC stains and suspended in the cell-free breast milk (Table 1). Aliquots of mock breast milk microbiota sample (1.5 mL) were stored at −80 °C for 1 week prior to analysis. Detailed bacterial culture methods are provided in supplementary methods.

**Table 1:** Details on bacterial strains cultured for mock breast milk sample, approximate CFU/mock sample, 16S rRNA gene copies/cell, and the expected copies of 16S rRNA genes/mock samples for each bacteria.

Bacteria for mock breast milk	McFarland Value	~CFU/ml	~16S rRNA gene copies/cell	~CFU/mock	~16S rRNA genes/mock
*Lactobacillus paracasei* (clinical isolate)	0.66	2 × 10^8^	5	3.3 × 10^5^	1.65 × 10^6^
*Bifidobacterium bifidum* (clinical isolate)	0.21	6 × 10^7^	4	1 × 10^5^	4 × 10^5^
*Streptococcus salivarius* ATCC 13419	0.66	2 × 10^8^	3	3.3 × 10^5^	9.9 × 10^5^
*Staphylococcus epidermidis* ATCC 14990	0.52	1.5 × 10^8^	5	1.5 × 10^5^	7.5 × 10^5^
*Enterococcus faecalis* ATCC 29212	0.68	2 × 10^8^	4	3.3 × 10^5^	1.32 × 10^6^
*Escherichia coli* ATCC 25922	0.67	2 × 10^8^	7	3.3 × 10^5^	2.31 × 10^6^

### DNA extraction methodologies

Five DNA extraction methods were assessed: DNeasy PowerLyzer PowerSoil DNA Isolation Kit (QIAGEN, Hilden, Germany); GenElute Bacterial Genomic DNA Kit (Sigma-Aldrich, Missouri, USA); QIAamp DNA Stool Mini Kit (QIAGEN, Hilden, Germany); QIAamp DNA Stool Mini Kit with the addition of bead-beating; and a manual phenol-chloroform based extraction method^54^. Methodologies are summarised in Table 2 and detailed in Supplementary Methods.

**Table 2:** Details of study design and the approach to lysis of each DNA extraction method used.

Method	Heat lysis	Chemical Lysis	Mechanical lysis
*DNeasy PowerLyzer PolerSoil DNA isolation (PS)*	65 °C 10 minutes	Buffers only (supplied)	Bead beating
*Sigma-Aldrich GenElute Bacterial Genomic DNA Kit (SA)*	55 °C 10 minutes	Lysis buffer and solution (supplied)	None
*Manual phenol-chloroform extraction method (MAN)*	95 °C 5 minutes	Lysostaphin and Lysozyme	Bead beating
*QIAamp DNA Stool Mini Kit (MK)*	95 °C 5 minutes	Buffers only (supplied)	None
*QIAamp DNA Stool Mini Kit with bead beating (MKBB)*	95 °C 5 minutes	Buffers only (supplied)	Bead beating

DNA extractions were run in parallel using the same laboratory perishables. Each method was applied to four aliquots of the mock breast milk sample and four aliquots of each of the human breast milk samples. Briefly, 1.5 mL of human breast milk or mock breast milk was pelleted by centrifuging at 13,000 *g*, 4 °C, 20 min. Fat layers from breast milk samples was removed along with liquid supernatant after cell pelleting. Following extraction, DNA was resuspended in a 50 μL volume of sterile DNase-free water or specified buffer (Supplementary Methods).

### DNA yield and purity

DNA yield was assessed using the Qubit dsDNA HS Assay kit with a Qubit 2.0 fluorometer (Life Technologies, Carlsbad, CA, USA). DNA purity was assessed based on the ratio of absorbance at 260 nm and 280 nm (A260/A280) using a Nanodrop spectrophotometer (NanoDrop Technologies, Wilmington, DE, USA). PCR amplification efficiency for respective extracts was further assessed by 16S rRNA gene quantitative PCR, as described previously (Denman *et al*. 2006). Detailed qPCR protocols are provided as Supplementary Methods.

### 16S rRNA gene amplicon sequencing

Amplicon sequencing of the V4 16S rRNA gene was performed as described previously^55^. Briefly, an initial PCR reaction was prepared that contained 11.5 µL of DNA, 5 μl of forward primer (1 mol/L), 5 μl of reverse primer (1 mol/L) and 12.5 μL of 2× KAPA HiFi Hotstart Ready Mix (KAPA Biosystems, Wilmington, Massachusetts) in a total volume of 25 μL. The PCR reaction was performed on a Veriti 96-well Thermal Cycler (Life Technologies) using the following program: 95 °C for 3 minutes, followed by 25 cycles of 95 °C for 30 seconds, 55 °C for 30 seconds, and 72 °C for 30 seconds, and a final extension step at 72 °C for 5 minutes. Samples were multiplexed using a dual-index approach with the Nextera XT Index kit (Illumina Inc., San Diego, CA, USA) according to manufacturer’s instructions. All DNA extracts were included in a single 16S rRNA sequencing run alongside a known mock community to account for potential PCR bias. The final library was paired-end sequenced at 2 × 300 bp using a MiSeq Reagent Kit v3 on the Illumina MiSeq platform (David R. Gunn Genomics Facility, South Australian Health and Medical Research Institute).

Overlapping paired-end reads 16S rRNA sequence reads were processed with DADA2 using QIIME2 (release 2018.2)^56,57^. Unique amplicon sequence variants (ASVs) were assigned a taxonomy and aligned to the SILVA v132 database trimmed to the V4 region of the 16S rRNA gene at 97% sequence similarity^58^. Microbial data was subsampled to a uniform depth of 1,553 reads. Detailed bioinformatic methods are provided in Supplementary Methods.

### Statistical analysis

Differences in yield, purity, and bacterial amplification were assessed by one-way ANOVA with Tukey’s Honest Significant Difference (HSD) or non-parametric Kruskal-Wallis one-way ANOVA with Dunn’s multiple comparison test, according to data distribution (Shapiro-Wilk statistic). Non-parametric analyses were performed using the GraphPad PRISM v7.03 (GraphPad Software Inc., California, USA) and analysis of parametric data were conducted using SPSS Statistics version 25.

Taxa diversity was described as richness (observed ASVs), diversity (Faith’s phylogenetic diversity), and evenness (Pielou’s evenness). Microbial dispersion of human breast milk samples was determined using the Weighted UniFrac distance matrix. These metrics were exported from QIIME2 core metrics output into PRIMER7 and inter-sample and method differences were tested by PERMANOVA. Nonmetric multidimensional scaling (nMDS) was performed to visualise the distances between methods.

Linear regression analysis was performed in GraphPad PRISM to investigate the relationship between DNA concentration and observed ASVs. R squared values and equation of the slope were used to demonstrate the line fit, with the data and the direction of the relationship with P < 0.05 considered significant. Taxa represented by spurious bacterial signal in sequence data generated from mock breast milk samples were visualised as a Krona plot^59^.

## Supplementary information

Supplementary Information 

Supplementary Information 2

## Data Availability

The raw sequence datasets generated and analysed in this study are available through the Sequence Read Archive under BioProject ID: PRJNA526772.
