# Supplementary material for: DNA extraction approaches substantially influence the assessment of the human breast milk microbiome
Source: Sci Rep. 2020 Jan 10;10:123. doi: 10.1038/s41598-019-55568-y (PMC6954186; doi:10.1038/s41598-019-55568-y)
Supplement: Supplementary file 1 — Supplementary Information [file 41598_2019_55568_MOESM1_ESM.docx]

**DNA extraction approaches substantially influence the assessment of the human breast milk microbiome**

Chloe A Douglas^1,2,3^, Kerry L Ivey^2,7^, Lito E Papanicolas^2^, Karen P Best^1^, Beverly S Muhlhausler^1,5,6^, Geraint B Rogers^2,4*^

**Supplementary Methods**

Bacterial culture from Mock Breast Milk sample

Clinical isolates of *Lactobacillus paracasei* and *Bifidobacterium bifidum* were plated on CHOC agar plates (ThermoFisher Scientific, Massachusetts, USA) and grown under anaerobic conditions at 37°C for 24 to 48 hours. *Streptococcus salivarius* (ATCC 13419), *Staphylococcus epidermidis* (ATCC 14990), *Enterococcus faecalis* (ATCC 29212) and Escherichia coli (ATCC 25922) were plated onto Horse Blood Agar (HBA) plates and grown under aerobic at 37°C for 24 to 48 hours. Single colonies were collected and resuspended in saline before being measured for their concentration (CFU/ml). Mcfarlands standard values were calculated using DENSICHEK (BioMérieux inc., Marcy-l'Étoile, France) to determine the approximate CFU/ml. Each bacteria was added in the same value and the estimated CFU/ml was calculated (Table 1).

16S rRNA gene qRT-PCR

The primers used were as follows; 1114F (5'-CGGCAACGAGCGCAACCC-3') and 1275R (5'-CCATTGTAGCACGTGTGTAGCC-3')[^58^](#_ENREF_58) which have a melting curve of 60°C. Thermal cycling was as follows; 50°C for 2 minutes and 95°C for 10 minutes, 40 cycles of 95°C for 15 seconds, 60°C for 1 minute, followed by melt curve analysis with an incremental temperature increase of 0.05°C every second from 60°C to 95°C on the QuantStudio 6 Flex Real-Time PCR System (ThermoFisher Scientific, Massachusetts, USA). Each reaction mixture was prepared in a 20µL volume consisting of the following components: 10 µL PowerUp SYBR Green Master Mix (ThermoFisher Scientific, Massachusetts, USA), 0.4 µL of each primer (10µm), 1 µL template, and 8.2 µL PCR water and run in 5µL volume triplicates with the addition of a NTC. Bacterial DNA concentration was calculated using a standard curve from a known concentration of Escherichia coli (ATCC 35218).

DNA extraction – DNeasy PowerLyzer PowerSoil DNA Isolation

DNA extraction using the DNeasy PowerLyzer PowerSoil DNA Isolation Kit (QIAGEN, Hilden, Germany) according to the manufacturer’s instructions with minor modifications: samples were eluted in 50 µL of dH2O rather than C6 solution. FastPrep-24 (MP Biomedicals, Santa Ana, USA) for homogenization and samples were homogenized for two pulses at 6.5m/s for 60 seconds.

The pellet was resuspended in Bead solution and added to PowerLyzer Glass Bead Tube, 0.1 mm with C1 solution and incubated at 65°C for 10 minutes. Samples were homogenised using the FastPrep-24 (MP Biomedicals, Santa Ana, USA) for 2 cycles at 6.5m/s for 60 seconds. The samples were spun down and supernatant collected into new tube with C2 solution. The samples were vortexed and incubated at 4°C for 10 minutes and spun down again. Supernatant was collected and added to a tube with C3 solution and then vortexed and incubated at 4°C for 10 minutes before being spun down. Supernatant was collected and added to a tube with C4 solution. Sample is then spun through the spin filter until all sample has passed through and all eluent has been discarded. C5 was added to the spin filter and eluent discarded. DNA was eluted in 50μl of dH2O (ThermoFisher Scientific, Massachusetts, USA).

DNA extraction – Sigma-Aldrich GenElute Bacterial Genomic DNA Kit

DNA extraction using the GenElute Bacterial Genomic DNA Kit (Sigma-Aldrich, Missouri, USA) according to the manufacturer’s instructions with minor modifications: samples were eluted in 50 µL of dH2O rather than Elution Solution (10 mM Tris-HCl, 0.5 mM EDTA, pH 9.0).

The pellet was resuspended in Lysozyme solution (Lysozyme in Gram-Positive Lysis Buffer) and incubated for 30 minutes at 37°C. Proteinase K and Lysis solution C were added to the sample and then incubated for 10 minutes at 55°C. The provided columns were prepared with the Preparation Solution and spun down, eluent is discarded. 100% ethanol was then added to the samples before added into the column and spun down, flowthrough is discarded. Column was then washed with the Wash Solution Concentrate and spun down until dry. DNA was eluted in 50μl of dH2O (ThermoFisher Scientific, Massachusetts, USA).

DNA extraction – Manual phenol-chloroform

DNA extraction using a manual phenol-chloroform method with the addition of bead beating and lysozymes for optimal lysis and extraction of gram positive bacteria.

The pellet was resuspended in 400μl of TE buffer (10nM Tris, 1mM EDTA; pH 8.0; Invitrogen) and incubated at 95°C for 5 minutes. Once cooled, Lysostaphin (Sigma-Aldrich, MO, USA) in phosphate buffered saline and Lysozyme (ROCHE, ThermoFisher Scientific, Massachusetts, USA) was added to the sample and inverted to mix. 5M Nacl was added and inverted to mix before incubated at 37°C for 1 hour. Proteinase K (20 mg/ml) (ThermoFisher Scientific, Massachusetts, USA) and 10% sodium dodecyl sulphate (SDS) (Sigma-Aldrich, MO, USA) was added and samples were incubated at 55°C for 30 minutes. 1.0 mm and 0.1 mm Zirconia/Silica Beads (BioSpec Products, Inc. OK, USA) were added to the sample and they were homogenised using the FastPrep-24 (MP Biomedicals, Santa Ana, USA) at 6.5m/s for 60 seconds. Sample was spun down and phenol:chloroform:isoamyl alcohol (25:24:1; saline buffered at pH8.0; Sigma-Aldrich, MO, USA) added and samples were vortexed to mix. Sample was centrifuged at 13,000g for 20 minutes at 4°C and top aqueous layer was collected. DNA was precipitated by the addition of 10M ammonium acetate in a 1:10 ratio and 100% ethanol (Sigma-Aldrich, MO, USA) in a 1:1 ratio with sample volume. Recovery of DNA was performed using an EZ-10 spin column as per the manufacturer’s instructions (Bio Basic Inc., Ontario, Canada) and eluted in 50μl UltraPure DNase/RNase-free distilled water (Gibco, ThermoFisher Scientific, Victoria, Australia).

DNA extraction - QIAamp DNA Stool Mini Kit

DNA extraction using the QIAamp DNA Stool Mini Kit (QIAGEN, Hilden, Germany) according to the manufacturer’s instructions (for hard to lyse bacteria).

The pellet was resuspended in Buffer ASL and samples were vortexed. The sample was incubated at 95°C for 5 minutes followed by briefly vortexing and centrifuged briefly. Supernatant was collected and an InhibitEX Tablet was added to each sample followed by vortexing and centrifuging for 3 minutes. Supernatant was collected and again centrifuged for 3 minutes. Collected supernatant was added to Proteinase K and Buffer AL. Samples were incubated at 70°C for 10 minutes. 100% ethanol was added to the samples and vortexed. The samples were added to the spin column and centrifuged for 1 minute, flowthrough discarded. Buffer AW1 was added and sample is again centrifuged for 1 minute, once flowthrough discarded this is repeated with Buffer AW2. DNA was eluted in 50μl of Buffer AE.

This method was repeated with the addition of bead beating after buffer ASL was added and sample were incubated at 95°C for 5 minutes. 1.0 mm and 0.1 mm Zirconia/Silica Beads (BioSpec Products, Inc. OK, USA) were added to the sample. Samples were homogenised using the FastPrep-24 (MP Biomedicals, Santa Ana, USA) at 6.5m/s for 60 seconds. The protocol was then completed as manufacturer instructions. DNA was eluted in 50μl of Buffer AE.

Sequence data bioinformatics

The Quantitative Insights In to Microbial Ecology software, QIIME 2 (release 2018.2), was used to analyse the 16S rRNA sequence generated from paired-end amplicon sequencing using the following pipeline. Overlapping paired-end reads 16S rRNA sequence reads were imported, converted to artifacts and demultiplexed [PairedEndSequencesWithQuality]. The quality of reads were visualised and truncation length chosen based on quality of the forward and reverse reads. Sequences were truncated, de-noised and merged using DADA2 [denoise-paired][^54^](#_ENREF_54)^,^[^55^](#_ENREF_55). Additionally, chimeric sequences were identified and removed via the consensus method in DADA2. Contaminating eukaryotic, mitochondrial and chloroplast sequences were filtered [taxa filter-table]. Reads were visualised [feature-table summarize] and subsampling depth was chosen according to the sample with the lowest reads. A phylogenetic tree was constructed for alpha and beta diversity measures using [phylogeny fasttree][^59^](#_ENREF_59)^,^[^60^](#_ENREF_60). Alpha and beta diversity measures are calculated using the input filtered table, phylogeny rooted tree, and chosen sub-sampling depth [diversity core-metrics-phylogenetic]. Once core metrics were produced [feature-classifier classify-sklearn], unique amplicon sequence variants (ASVs) were assigned a taxonomy and aligned to the SILVA v132 database trimmed to the V4 region of the 16S rRNA gene at 97% sequence similarity [metadata tabulate][^56^](#_ENREF_56). ASVs clustered at 97% similarity and box plots are output [taxa barplot]. Microbial data was subsampled to a uniform depth of 1,553 reads.

**Supplementary figure legends**

**Supplementary Figure 1.** Sequencing reads per sample after demultiplexing for the mock breast milk (A) and human breast milk samples (B). Significant of the mock breast milk (A) was determined by one-way ANOVA with Tukey's Honest Significant Difference. Significant of human breast milk samples (B) was determined by non-parametric Kruskal-Wallis ANOVA with Dunn’s multiple comparison correction. * = P<0.05, ** = P<0.01, *** = P<0.001, **** = P<0.0001. PS = DNeasy PowerLyzer PowerSoil DNA Isolation, SA = Sigma-Aldrich GenElute Bacterial Genomic DNA Kit, MAN = manual phenol-chloroform extraction method, MK = QIAamp DNA Stool Mini Kit, MKBB = QIAamp DNA Stool Mini Kit with bead beating.

**Supplementary Figure 2.** Mean relative abundance of spurious bacteria across all methods in mock breast milk sample.

**Supplementary Figure 3.** Alpha diversity measures for human breast milk samples. Significant was determined by non-parametric Kruskal-Wallis ANOVA (P<0.05), with Dunn’s multiple comparison correction. * = P<0.05, ** = P<0.01, *** = P<0.001, **** = P<0.0001.PS = DNeasy PowerLyzer PowerSoil DNA Isolation, SA = Sigma-Aldrich GenElute Bacterial Genomic DNA Kit, MAN = manual phenol-chloroform extraction method, MK = QIAamp DNA Stool Mini Kit, MKBB = QIAamp DNA Stool Mini Kit with bead beating.

**Supplementary Figure 4.** Linear regression analysis of the relationship between DNA concentration (ng/µl) and ASVs (A), relative abundance of *Delftia* (B), *Flavobacterium* (C), *Pseudomonas* (D), *Staphylococcus* (E), and *Streptococcus* (F). PS = DNeasy PowerLyzer PowerSoil DNA Isolation, SA = Sigma-Aldrich GenElute Bacterial Genomic DNA Kit, MAN = manual phenol-chloroform extraction method, MK = QIAamp DNA Stool Mini Kit, MKBB = QIAamp DNA Stool Mini Kit with bead beating.

**Supplementary Figure 5.** Linear regression analysis of the relationship between DNA concentration (ng/µl) of human breast milk samples and relative abundance of *Delftia* (A), *Flavobacterium* (B), *Pseudomonas* (C), *Staphylococcus* (D), and *Streptococcus* (E). PS = DNeasy PowerLyzer PowerSoil DNA Isolation, SA = Sigma-Aldrich GenElute Bacterial Genomic DNA Kit, MAN = manual phenol-chloroform extraction method, MK = QIAamp DNA Stool Mini Kit, MKBB = QIAamp DNA Stool Mini Kit with bead beating.

**Supplementary Figure 1**

**
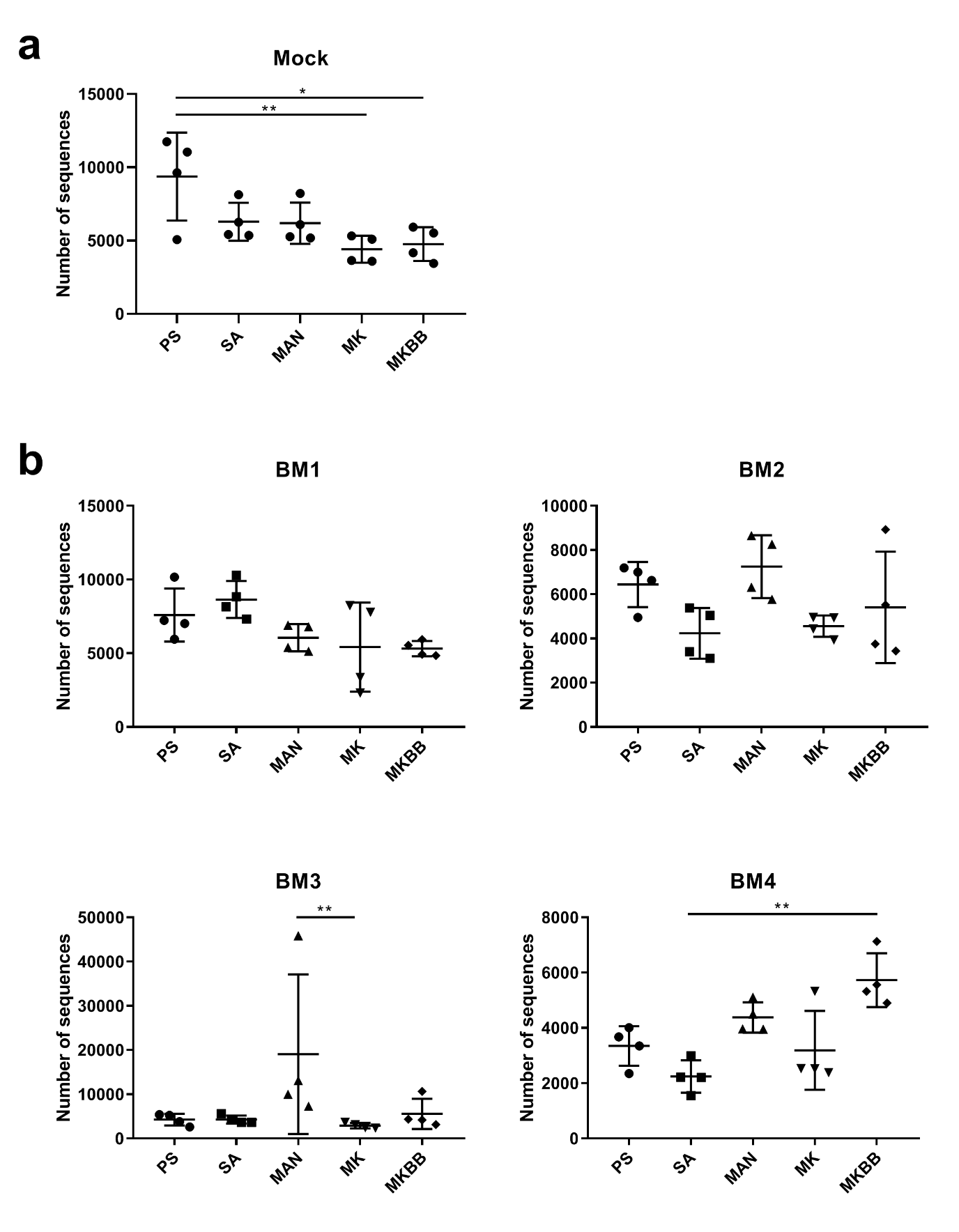
**

**Supplementary Figure 2**

**
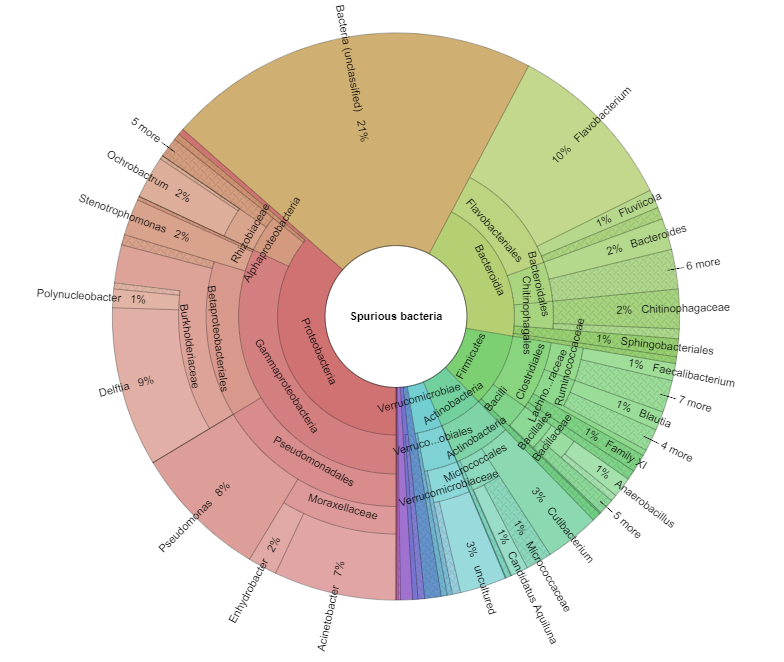
**

**Supplementary Figure 3**

**
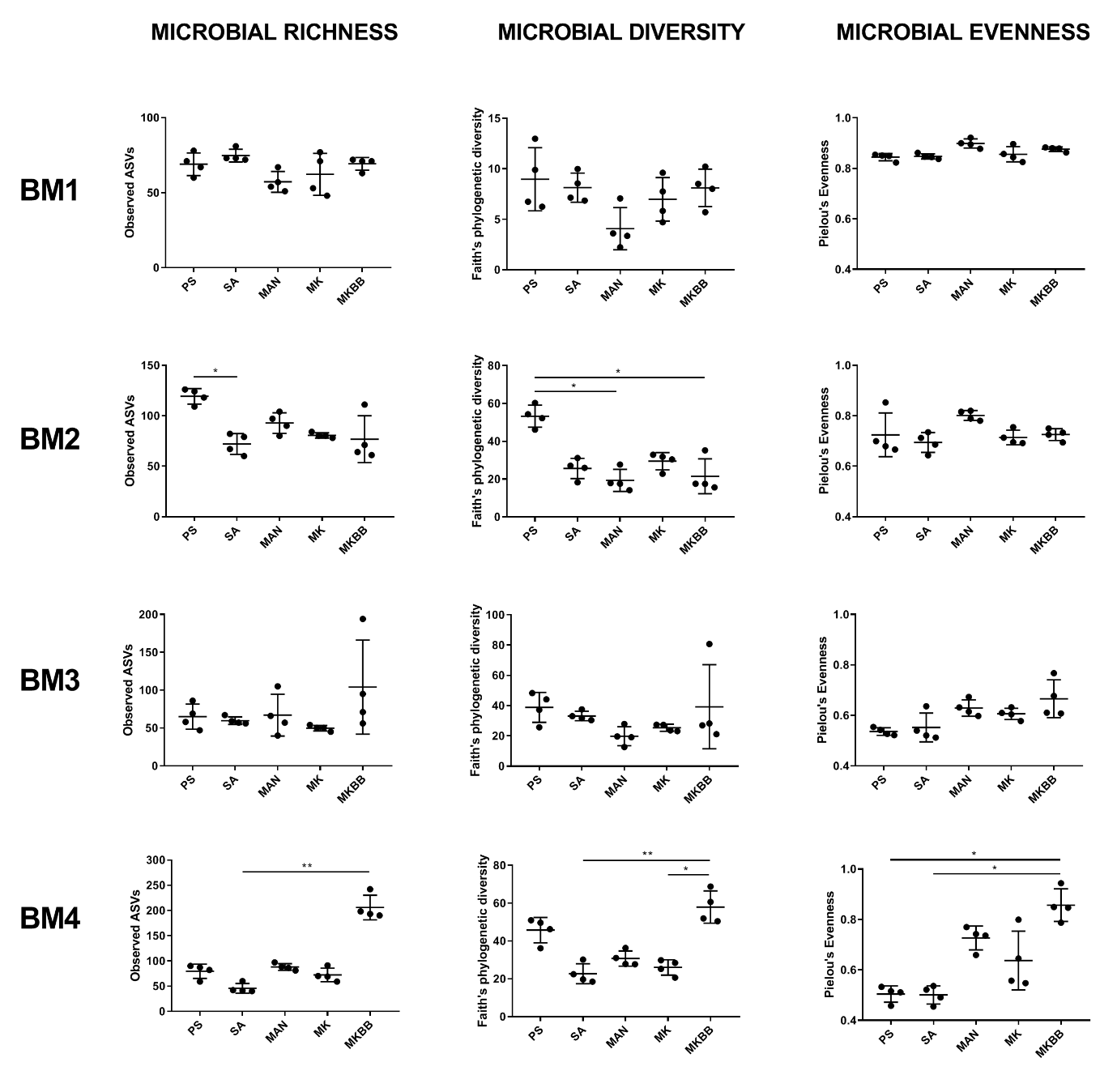
**

**Supplementary Figure 4**

**
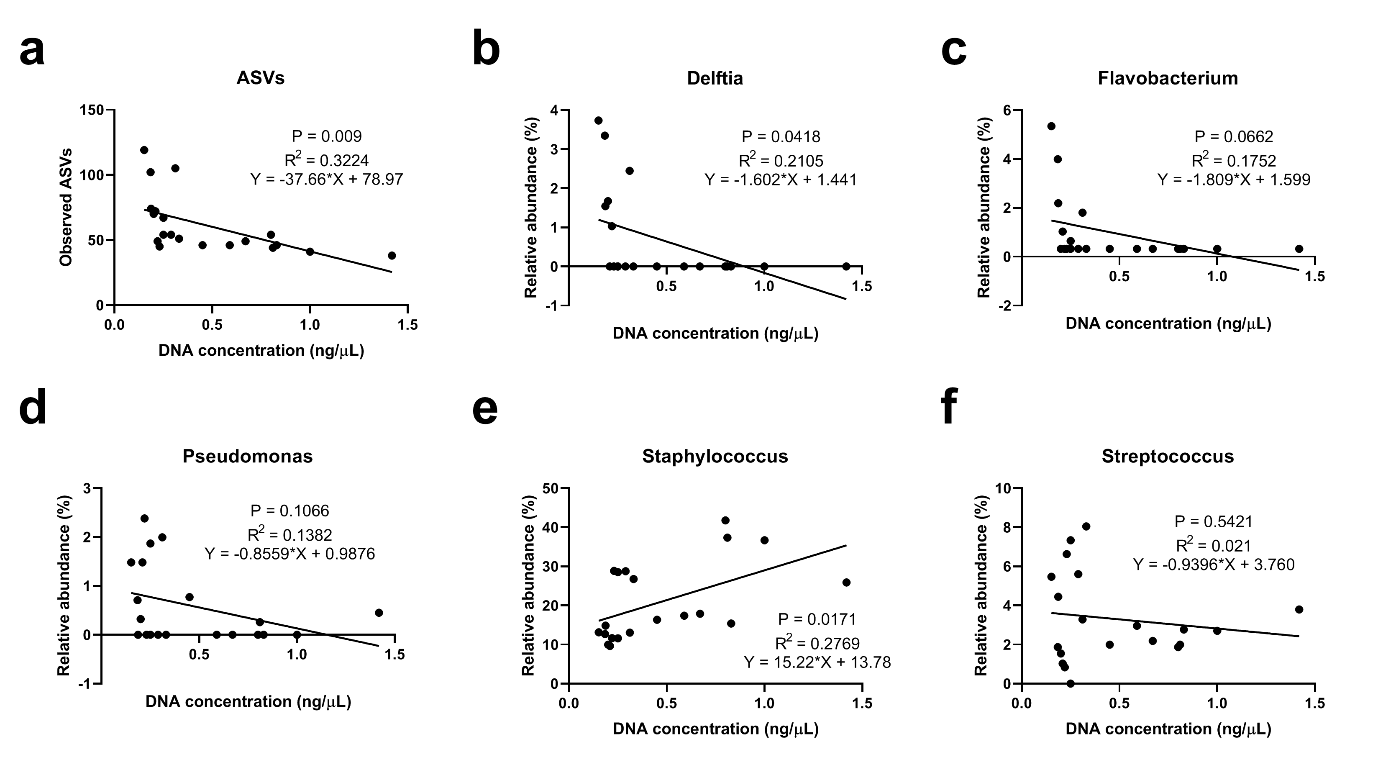
**

**Supplementary Figure 5**

**
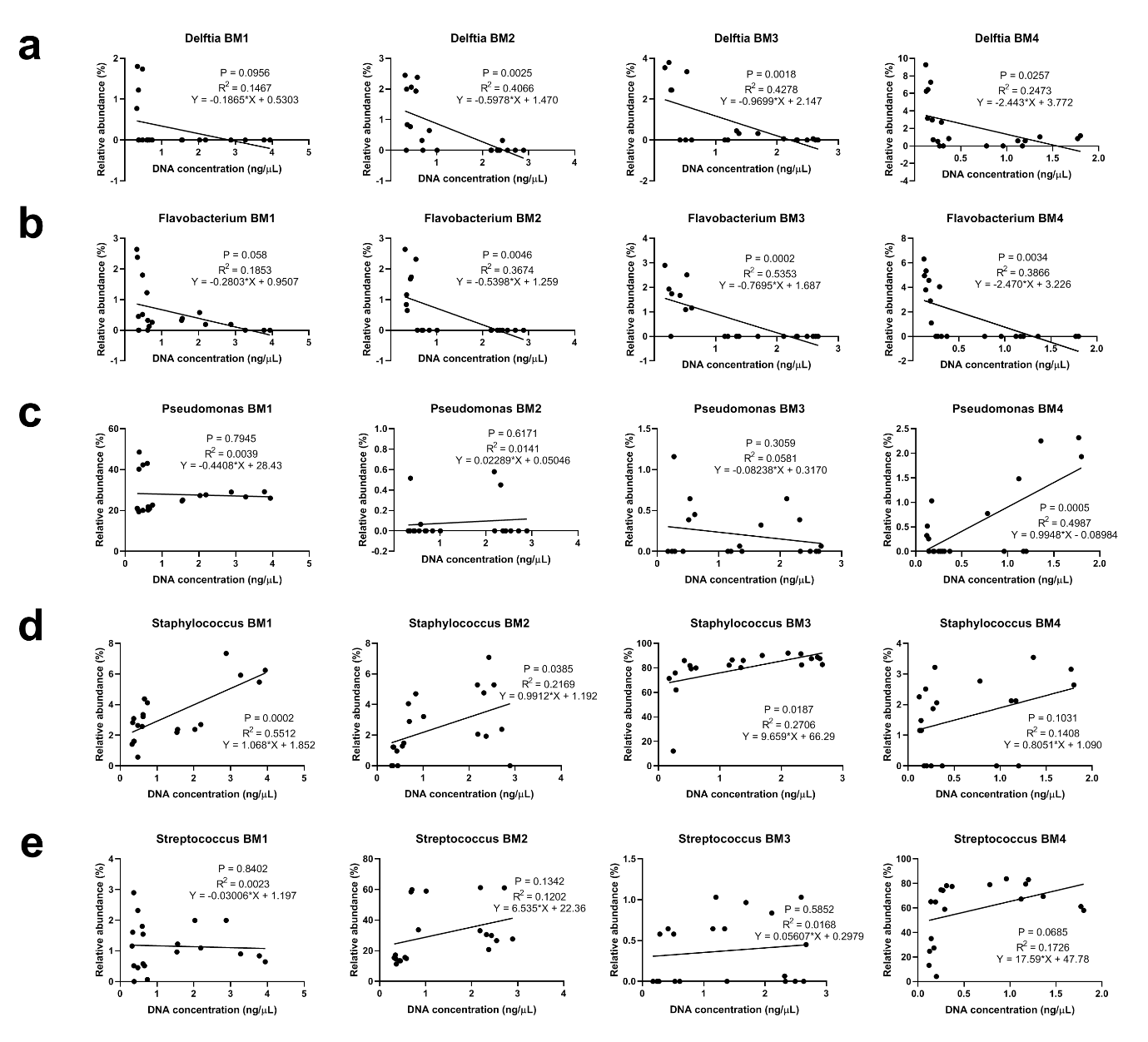
**
